# Antenatal Indomethacin Treatment for Congenital Myotonic Dystrophy

**DOI:** 10.1155/2019/4290145

**Published:** 2019-02-14

**Authors:** Kyohei Yamaguchi, Hiroaki Tanaka, Fumi H. Furuhashi, Kayo Tanaka, Eiji Kondo, Tomoaki Ikeda

**Affiliations:** Department of Obstetrics and Gynecology, Mie University, Mie, Japan

## Abstract

Myotonic dystrophy is an autosomal-dominant disorder. Its congenital type is the most severe form, with respiratory failure that can be a life-threatening event after birth. There are no antenatal treatments that can improve neonatal outcomes of myotonic dystrophy. We treated a fetus with congenital myotonic dystrophy by administering indomethacin to the 31-year-old Japanese mother affected by myotonic dystrophy and polyhydramnios. We observed increased fetal breathing movement and a reduction of the amniotic fluid volume. The baby was born at 37 weeks and discharged from the neonatal intensive care unit with a favorable outcome. Indomethacin treatment is likely to improve fetal lung function and to control the amniotic fluid volume. This report emphasizes the importance of further investigations regarding the optimal management of congenital myotonic dystrophy.

## 1. Introduction

Myotonic dystrophy (DM) is a common autosomal-dominant systemic condition characterized by various symptoms including muscular weakness and atrophy, myotonia, cardiac conduction defects, temporal balding, mental retardation, and various endocrinopathies [1]. The incidence of DM is estimated to be 1 in 8,000 adults. Congenital myotonic dystrophy is the most severe form of DM. Genetically, DM is caused by the expansion of an unstable CTG sequence in the myotonic protein kinase (DMPK) gene at chromosome 19q13.3 [2]. The expansion is observed in parent to child transmission, which is called “anticipation.” The anticipation is more frequent and critical when the inheritance occurs from the mothers rather than the fathers [3]. Respiratory failure is the most critical complication of congenital DM, resulting in a high mortality rate in the neonatal period [4]. There are no effective antenatal treatments for a fetus with congenital DM at this time.

We present the case of a fetus with congenital DM who we treated with antenatal indomethacin (which is thought to stimulate fetal breathing movement), with a favorable outcome. We hope this report will encourage further investigations into the optimal treatments for congenital DM.

## 2. Case Presentation

A 31-year-old primipara Japanese patient by natural conception with a history of miscarriage was referred to our department at 24 weeks of gestation because of polyhydramnios. The amniotic fluid index (AFI) was 32 cm, and there was no evidence of diabetes mellitus or fetal neurologic impairment. Ultrasound examinations showed decreased fetal body movement without any fetal structural anomalies.

At 28 weeks of gestation, blood sampling revealed that the patient's serum creatine phosphokinase (CPK) level was markedly high at 7136 U/L, accompanied by lower-body muscular pain. The physical and neurological examinations showed grip myotonia and percussion myotonia with hatchet face. The patient was diagnosed as having myotonic dystrophy based on electromyographic investigations. DNA testing at 30 weeks and 4 days of gestation revealed an expansion of 1100–1200 abnormal CTG repeats within the myotonin protein kinase gene. The patient's family history was not known.

**Figure 1:**
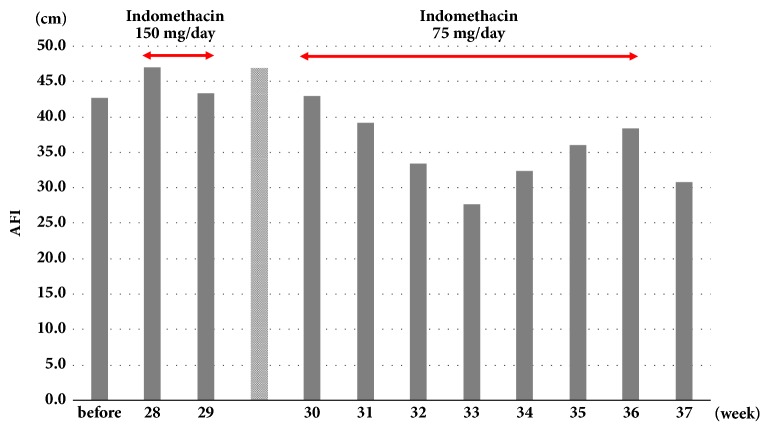
Amniotic fluid index (cm). The data are weekly averages. The data in the gray bar are corrected at 29 weeks and 6 days of gestation.

**Figure 2:**
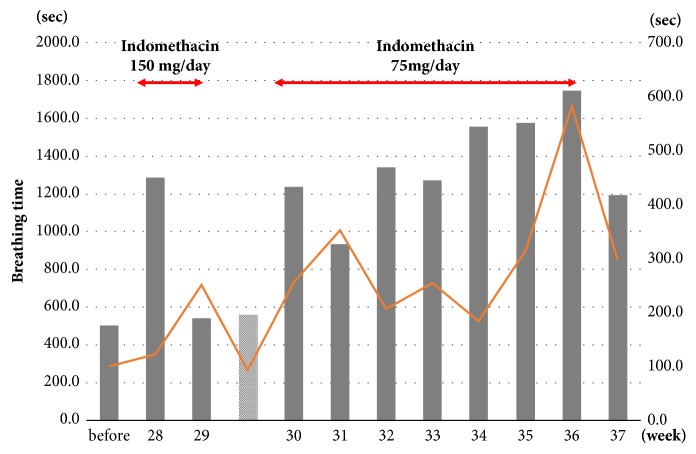
Breathing time (sec). The data are weekly averages. The left vertical scale is the total breathing time (shown in the black bar) in 30 min. The right vertical scale is each average breathing time (the orange line). The data in the gray bar are corrected at 29 weeks and 6 days of gestation.

We hypothesized that the prostaglandin inhibitor indomethacin could improve the fetus' lung function. A rectal 150 mg/day of indomethacin was administered to the mother from 28 weeks and 4 days of gestation, and weekly serum sampling and daily ultrasound examinations (at 2:00–3:00 pm) were performed. Fetal breathing movement lasting more than 30 seconds in 30 minutes was counted and total breathing time was measured. However, at 29 weeks and 4 days of gestation the indomethacin administration was stopped temporarily due to constriction of the fetal ductus arteriosus (0.6 mm dia.) [5] and severe tricuspid regurgitation. Thereafter, a rectal 75 mg/day of indomethacin was administered to the mother until 37 weeks of gestation, and the fetal ductal arteriosus returned to a normal value thereafter. The AFI had not increased through the course of the treatment (Figure 1), and no signs of threatened labor were observed. During the indomethacin treatment, a tendency for an increase in both the total breathing time and each average breathing time in 30-min ultrasonographic examinations was observed (Figure 2).

At 37 weeks and 6 days of gestation, we performed an elective cesarean with spinal anesthesia because of the abnormal fetal position. The patient required the support of biphasic positive airway pressure for 24 hr after the operation. The total blood loss was 1.8 L (including amniotic fluid), but the patient did not need any blood transfusion and there were no signs of uterine atony. The patient was discharged 7 days after the operation without any complications.

The female infant's body weight was 2838 g and the Apgar scores were 5 and 6 at 1 and 5 min, respectively. Umbilical cord blood gas studies showed normal values. The newborn was markedly hypotonic with weakness of breathing, and she required intubation for 2 days and oxygen support until day 9. The ductus arteriosus closed at day 23. A physiological examination showed distal dominant hypotonia and swallowing dysfunction, but no arrhythmia, endocrinopathies, cataracts, or sensory-neural hearing loss was observed. A genetic test was performed at day 10 confirming congenital DM by expansion of 1300–1450 abnormal CTG repeats. The baby was discharged from the hospital at day 49.

## 3. Discussion

Our patient's case provides two important clinical implications. The most striking finding is that an antenatal rectal 75 mg/day of indomethacin has the potential benefit of safely improving fetal breathing movement. Additionally, indomethacin may be a useful drug for preventing threatened preterm labor in patients with DM.

Fetal breathing movements play an important role in lung growth and maturation by the cyclic expansion and retraction of the pulmonary parenchyma. The lung dysfunction of congenital DM fetus comes from both defect of respiratory control system and respiratory muscle function. There is much evidence that prostaglandins (especially PGE2) are responsible for the inhibition of breathing activity, which is controlled mainly at the brainstem [6, 7], since the inhibition of prostaglandins by indomethacin stimulated continuous breathing movement in an animal fetal model [8]. One prospective, randomized, placebo-controlled clinical trial demonstrated that a single dosage of indomethacin (50 mg) increases fetal breathing movement [9]. Although fetal breathing movement becomes episodic and its amplitude deepens with maturation [10], the present case showed an apparent trend of the improvement of both the single-measurements' average and the 30-min total fetal breathing movement.

Indomethacin, a nonsteroidal anti-inflammatory medication that inhibits prostaglandin production, freely crosses the placenta and enters fetal circulation. It is widely used as a tocolytic drug generally limited to short courses of therapy (48–72 h) [11]. However, the optimal dose and course of indomethacin have not been established and remain controversial.

Constriction of the fetal ductus arteriosus is a well-recognized side effect of indomethacin, and tricuspid regurgitation is caused by elevated pressure in the right ventricular outflow tract, producing mild endocardial ischemia with papillary muscle dysfunction. However, these side effects are usually transient and reversible when indomethacin therapy is stopped at an adequate time before delivery. In the present case, the constriction of the fetal ductus arteriosus (0.6 mm dia.) [5] and severe tricuspid regurgitation at 29 weeks and 4 days were resolved after the total rectal indomethacin dose was reduced to 75 mg/day.

Idiopathic polyhydramnios is one of the important expressions of congenital DM that leads to threatened preterm labor. Physiologically, idiopathic polyhydramnios is considered a consequence of reduced or absent fetal swallowing associated with impaired pharyngo-esophageal motility. Unfortunately, there are no useful tocolytic agents for pregnant women with DM because of its side effects such as rhabdomyolysis [12] and maternal respiratory compromise; [13] therefore, indomethacin treatment is recommended instead.

Our present patient similarly presented polyhydramnios during the 3rd trimester as in previous studies [4, 14] and as we had expected, the amniotic fluid volume gradually decreased as a result of reduced fetal urine output without any signs of threatened preterm labor. However, the relative increase of amniotic fluid volume in the late preterm period was unclear, and presumably this reflects the complex mechanisms of the regulation of amniotic fluid volume in utero [15] and depressed swallowing of the affected fetus.

To the best of our knowledge, this is the first report of the indomethacin treatment of a fetus with congenital DM with the goal of ameliorating fetal lung function and controlling the amniotic fluid volume, which eventually helped prevent threatened preterm labor.

We conclude that indomethacin may be an appropriate therapeutic drug for a fetus with congenital DM, as long as careful monitoring and management are performed. Further studies are needed to establish the safe dose and optimal course for indomethacin administration to a fetus with congenital DM.
